# Small for gestational age and anthropometric body composition from early childhood to adulthood: the Aboriginal Birth Cohort study

**DOI:** 10.3389/fpubh.2024.1349040

**Published:** 2024-02-21

**Authors:** Craig Hansen, Belinda Davison, Gurmeet R. Singh

**Affiliations:** ^1^Menzies School of Health Research, Charles Darwin University, Darwin, NT, Australia; ^2^Northern Territory Medical Program, Flinders University, Darwin, Darwin, NT, Australia

**Keywords:** small for gestational age, body composition, Indigenous Australian, anthropometrics, remote Australian communities

## Abstract

**Background:**

In Australia the estimated rate of small for gestational age (SGA) births is 9% among non-Indigenous births compared to 14% among Aboriginal and Torres Strait Islanders. There is limited research investigating the effect of being born SGA on body composition later in life in Indigenous Australians.

**Methods:**

Using data from the Aboriginal Birth Cohort longitudinal study, we compared the body composition of those born SGA to non-SGA by analysing anthropometric measures (height, weight, waist circumference, fat percentage [FAT%], body mass index [BMI], waist-to-height ratio, and A body shape index [ABSI]) collected at four follow-up periods (from childhood to adult). For cross-sectional analyses, linear regression models were employed to assess factors associated with anthropometric measures. For longitudinal analyses linear mixed models were employed to assess differences in anthropometric measures among SGA versus non-SGA individuals while adjusting for repeated measures.

**Results:**

The analytic baseline cohort were those who participated in Wave 2 (*n* = 570). In cross-sectional analyses, across all waves those born SGA had smaller anthropometric z-scores compared to non-SGA individuals (β ranging from −0.50 to −0.25). Participants residing in urban environments were significantly larger in Waves 2 to 4 (β ranged 0.26 to 0.65). Those born SGA had higher ABSI scores in Waves 2 and 4 (β 0.26 and 0.37, respectively). In longitudinal analyses, those born SGA had smaller measures of body composition across the life course; these differences were larger in urban communities. In remote communities those born SGA had significantly higher ABSI scores during adolescence and young adulthood, and this difference was not observed in urban communities.

**Conclusion:**

Indigenous Australians born SGA are smaller anthropometrically later in life compared to their non-SGA counterparts. In remote communities, those born SGA had higher levels of central adiposity compared to non-SGA.

## Introduction

Small for gestational age (SGA) is defined as a birth weight < 10th centile for gestational age, and those born SGA have an elevated risk of chronic diseases in adulthood, setting the stage for lifelong health disparities (1, 2). In Australia, the estimated rate of SGA births is approximately 9% in non-Indigenous and 14% among Aboriginal and Torres Strait Islander (hereafter, respectively, referred to as Indigenous Australians) births (3). Higher rates of SGA births are reported in remote Australian Indigenous communities (4) with comparable rates to low-to-middle income countries (5, 6).

The result of SGA followed by rapid weight gain during early postnatal life has been associated with increased long-term risks for central obesity, insulin resistance, impaired glucose tolerance, type 2 diabetes, hypertension, increased fat mass, and cardiovascular disease.

SGA is an adaptation to a less than ideal intrauterine environment. A period of catch-up growth occurs between 6 months and 2 years of age, followed by a trajectory of typical growth. However, SGA babies tend to be shorter and lighter than their appropriate for gestational age (AGA) cohorts (1, 2). The weight gain experienced by SGA babies typically has less fat-free body mass than AGA babies, hypothesized due to a consequence of the mismatch between the adaptations for survival *in-utero* and the abundant postnatal nutritional environment. This mismatch forms the basis of increased risk of central obesity, insulin resistance, compromised glucose tolerance, type 2 diabetes, hypertension, elevated fat mass, and cardiovascular disease seen in infants born SGA who undergo rapid weight gain during early postnatal life (2, 7–10). Obesity is a major risk factor for adult chronic diseases and the combination of SGA and later obesity amplifies this risk. Two studies analysing data from The Study of Longitudinal Indigenous Children (1,759 children born 2001–2008) reported that BMI is significantly lower among those categorised as being moderate-to-high prenatal risk (derived from gestational age, SGA, and birth weight) compared to those born full-term (11); and increased birth weight is associated with increased childhood BMI (12).

The Aboriginal Birth Cohort (ABC) is an Australian prospective longitudinal study investigating the long-term impact of early life factors on health and the burden of disease among Indigenous communities. The ABC study is the longest-running and largest Indigenous birth cohort in Australia with follow-up data collected over three decades among a cohort of 686 babies born to Indigenous Australian mothers. When first followed up at 11 years of age (Wave 2), and subsequently at 18 years of age (Wave 3), individuals born SGA remained significantly smaller anthropometrically compared to their non-SGA peers at both timepoints (13, 14), suggesting limited catch-up growth among those born SGA. The ABC cohort have since been followed up two more times, aged at 23–28 years (Wave 4) and 29–36 years (Wave 5). Recent research analysed anthropometric measures from Waves 2, 3, and 4 in association with socioeconomic status and remoteness, however SGA status was not part of the analyses (15).

The current study aims to extend the previous work of Sayers et al. (13, 14) by examining changes in anthropometric measures, including measures of fat, across the entire life-course comparing SGA and non-SGA individuals living in both urban and remote communities.

## Methods

### Study design

The ABC study is a prospective longitudinal cohort where data has been collected at birth and across four subsequent waves (birth to the age of mid-thirties). For the current study, we utilise both cross-sectional and longitudinal study designs. For the cross-sectional design we analyse data within each study wave separately, and for the longitudinal design we analyse data from all waves combined.

### Setting and study participants

The data analysed in this study come from the ABC study and the recruitment methods have been reported in detail elsewhere (16). Briefly, 686 babies born to Aboriginal mothers at the Royal Darwin Hospital from 1987 to 1990 were recruited for the study. During that period the Royal Darwin Hospital served as the primary facility for Indigenous mothers, attracting over 90% of pregnant Indigenous mothers from a region spanning 400,000 km^2^ in the “Top End” of the Northern Territory. Notably, 75% of the study cohort lived in remote communities, including the Arnhem, Victoria Daly, and Tiwi regions, while 25% resided in urban communities, including Darwin and its immediate surroundings.

**Figure 1:**
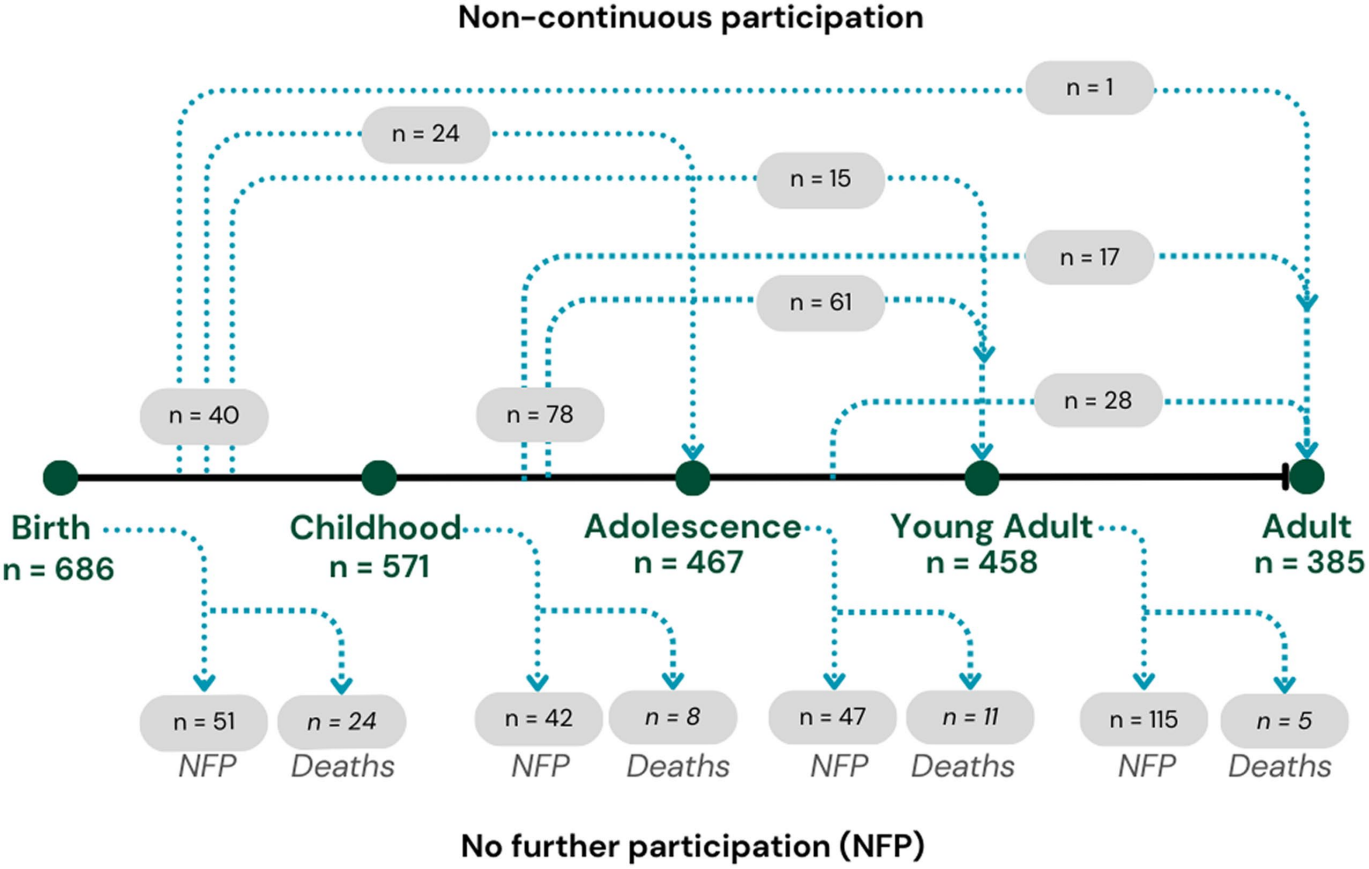
Life course participation numbers in the Aboriginal Birth Cohort study across waves 1 to 5.

Throughout their life course, the ABC cohort have been followed-up four times (Wave 2: at childhood aged 8–14 years; Wave 3: at adolescence aged 16–21 years; Wave 4: at young adult aged 23–28 years; and Wave 5: at adult aged 29–36 years). The ABC study recruited 686 Indigenous births (Wave 1) with 385 participating 36 years later (Wave 5), resulting in 301 participants being lost to follow-up during the study period, with 38 of these lost to death. As shown in Figure 1, study participants were sourced for follow-up at each wave regardless of participation in previous waves, resulting in non-continuous participation for some of the cohort. The anthropometric measures of interest were first collected at Wave 2, and therefore the baseline analytic cohort for the current study are those who participated in Wave 2 (*n* = 570 and excluding 1 participant in a wheelchair). The analyses include data from all subsequent waves, regardless of non-continuous participation.

### Variables of interest

#### Demographic information

The demographic variables collected at each wave and analysed in this study were sex (male, female), age, location (remote, urban), and lifestyle factors smoking (yes, no), and alcohol use (yes, no) collected in Waves 3, 4, and 5.

#### Anthropometric measures (outcomes of interest)

The methods used to record anthropometric measures in the ABC study have been reported elsewhere (13, 14, 17). Briefly, in each phase of the ABC study, trained researchers assessed participants’ body size and shape while they wore lightweight clothing and were barefoot. Height measurements were recorded to the nearest millimetre using a portable stadiometer on a flat, hard surface. Weight and lean mass were measured to the nearest 0.1 kg using a digital electronic scale (TBF-521, Tanita Corporation, Illinois, United States) and assessed through bioimpedance analysis. Waist circumference (WC) was measured in centimetres (cm) at a horizontal plane, midway between the lowest ribs and the iliac crests. For the current study, the following indices and ratios were computed: Body mass index (BMI) was calculated using the standard formula (weight[kg]/height[m]^2), fat percentage (FAT%) was derived by dividing fat mass by weight, waist-to-height ratio (WHtR) was calculated by dividing waist circumference by height, and the “A body shape index” (ABSI) was computed using the formula (WC/BMI^2/3 * Height^1/2). The ABSI, a relatively recent index developed by Krakauer and Krakauer (18), was employed as a measure of central obesity independent of BMI.

#### Small for gestational age (predictor of interest)

Measures of birth weight and length, and gestational age estimations taken at birth, have previously been described in detail (13). Small for gestational age (SGA) was defined as those with a birth weight < 10th centile for gestational age, and non-SGA as those with a birth weight ≥ 10th centile for gestational age.

### Statistical analyses

Descriptive statistics, including frequencies, percentages, and means with standard deviations, are reported for demographic information and anthropometric measures. Analyses to compare anthropometric measures between non-SGA and SGA individuals were conducted both cross-sectionally and longitudinally. Cross-sectional analyses, within each wave, employed linear regression models to examine associations between anthropometric measures standardised as z-scores (calculated internally within each wave cohort), and potential predictors of sex (reference = male), age, SGA status (reference = non-SGA), and geographic location (reference = remote). All predictors were included in the models simultaneously. Smoking (yes/no) and alcohol (yes/no) were not collected at Wave 2 and therefore only included in the cross-sectional analyses for Waves, 3, 4, and 5. We additionally explored an interaction term involving SGA status and residential location to assess cross-differences in these groups.

Longitudinal analyses used linear mixed models to assess changes in anthropometric measures over time and differences between SGA and non-SGA and remote and urban residents. Random intercepts and slopes were included for each study participant to account for repeated measures (19). All anthropometric measures were standardised as z-scores (calculated internally across all waves combined) and were entered into models as outcomes regressed on sex (reference = male), age (with a polynomial cubed term), SGA status (reference = non-SGA), and geographic location (reference = remote). We present the results from these models two different ways: (1) plotting of the estimated marginal means for each age throughout the study period, categorised by SGA status and residential location and (2) post-hoc contrasts (e.g., differences in marginal means) between SGA and non-SGA estimated at ages 11, 18, 25, and 32 years (median age in each wave respectively), stratified by residential location.

All data preparation and analyses were performed using R version 4.2.2 (20). Linear mixed models were conducted using the lme4 package (21) and model contrasts and plotting of estimated marginal means were performed using the emmeans package (22).

## Results

### Descriptive characteristics

The baseline analytic cohort for this study are those who completed Wave 2 (*n* = 570), and of these, 443 (77.7%) completed Wave 3, 426 (74.7%) completed Wave 4, and 363 (63.7%) completed Wave 5 (Table 1). Of the 570 in the baseline cohort, 444 (77.9%) were born in remote areas and 125 (21.9%) in urban areas. At Wave 2 there were 424 (74.4%) residing in remote areas, increasing to 85.3% residing in remote areas at Wave 5 (chi square 15.54, *p = 0.001*). Of the 570 in the baseline cohort, 115 (20.2%) were born SGA with the participation across waves remaining consistent (chi square 0.168, *p = 0.982*). The percentage of female participants increased slightly across the waves (Wave 2 = 46.6% to Wave 5 = 49.9%), however this was not statistically significant (chi square 2.39, *p = 0.496*). During Waves 3 to 5 there were 45 pregnancies belonging to 42 distinct females, which were excluded from analyses for the wave they were pregnant. At Wave 4, two of the pregnancies were multiparous and at Wave 5 one pregnancy was multiparous.

**Table 1:** Descriptive characteristics of the study cohort from childhood to adult, stratified by small for gestational age (SGA) at birth.

	Wave 2 (Aged 8–14 yrs.)			Wave 3 (Aged 16–21 yrs.)			Wave 4 (Aged 23–28 yrs.)			Wave 5 (Aged 29–36 yrs.)		
	SGA	Non-SGA	All	SGA	Non-SGA	All	SGA	Non-SGA	All	SGA	Non-SGA	All
Total (*n*, row %)	115 (20.2)	455 (79.8)	570	89 (20.1)	354 (79.9)	443	82 (19.2)	344 (80.8)	426	71 (19.6)	292 (80.4)	363
Sex (*n*, column %)												
Male	60 (52.2)	243 (53.4)	303 (53.2)	40 (44.9)	182 (51.4)	222 (50.1)	36 (43.9)	170 (49.4)	206 (48.4)	38 (53.5)	145 (49.7)	183 (50.4)
Female	55 (47.8)	212 (46.6)	267 (46.8)	49 (55.1)	172 (48.6)	221 (49.9)	46 (56.1)	174 (50.6)	220 (51.6)	33 (46.5)	147 (50.3)	180 (49.6)
Pregnant^a^	0	0	0	6 (6.7)	14 (4.0)	20 (4.5)	6 (13.0)	15 (8.6)	21 (9.0)	0	4 (2.7)	4 (1.1)
Birth location												
Remote	95 (82.6)	349 (76.7)	444 (77.9)	74 (83.1)	285 (80.5)	359 (81.0)	68 (82.9)	282 (82.0)	350 (82.2)	60 (84.5)	251 (86.0)	311 (85.7)
Urban	20 (17.4)	106 (23.3)	125 (21.9)	15 (16.9)	69 (19.5)	84 (19.0)	14 (17.1)	62 (18.0)	76 (17.8)	11 (15.5)	41 (14.0)	52 (14.3)
Wave location												
Remote	89 (77.4)	335 (73.6)	424 (74.4)	73 (82.0)	276 (78.0)	349 (78.8)	65 (79.3)	265 (77.0)	330 (77.5)	63 (88.7)	245 (83.9)	308 (84.8)
Urban	26 (22.6)	120 (26.4)	146 (25.6)	16 (18.0)	78 (22.0)	94 (21.2)	17 (20.7)	79 (23.0)	96 (22.5)	8 (11.3)	47 (16.1)	55 (15.2)
Smoking												
No	–	–	–	29 (32.6)	92 (26.0)	121 (27.3)	18 (22.0)	85 (24.7)	103 (24.2)	20 (28.2)	75 (25.7)	95 (26.2)
Yes	–	–	–	45 (50.6)	216 (61.0)	261 (58.9)	52 (63.4)	217 (63.1)	269 (63.1)	47 (66.2)	205 (70.2)	252 (69.4)
Missing	115 (100)	455 (100)	570 (100)	15 (16.9)	46 (13.0)	61 (13.8)	12 (14.6)	42 (12.2)	54 (12.7)	4 (5.6)	12 (4.1)	16 (4.4)
Alcohol												
No	–	–	–	48 (53.9)	188 (53.1)	236 (53.3)	31 (37.8)	145 (42.2)	176 (41.3)	37 (52.1)	128 (43.8)	165 (45.5)
Yes	–	–	–	32 (36.0)	144 (40.7)	176 (39.7)	41 (50.0)	161 (46.8)	202 (47.4)	30 (42.3)	152 (52.1)	182 (50.1)
Missing	115 (100)	455 (100)	570 (100)	9 (10.1)	22 (6.2)	31 (7.0)	10 (12.2)	38 (11.0)	48 (11.3)	4 (5.6)	12 (4.1)	16 (4.4)

a Percentage based on total females in that cell.

### Cross-sectional analyses

**Figure 2:**
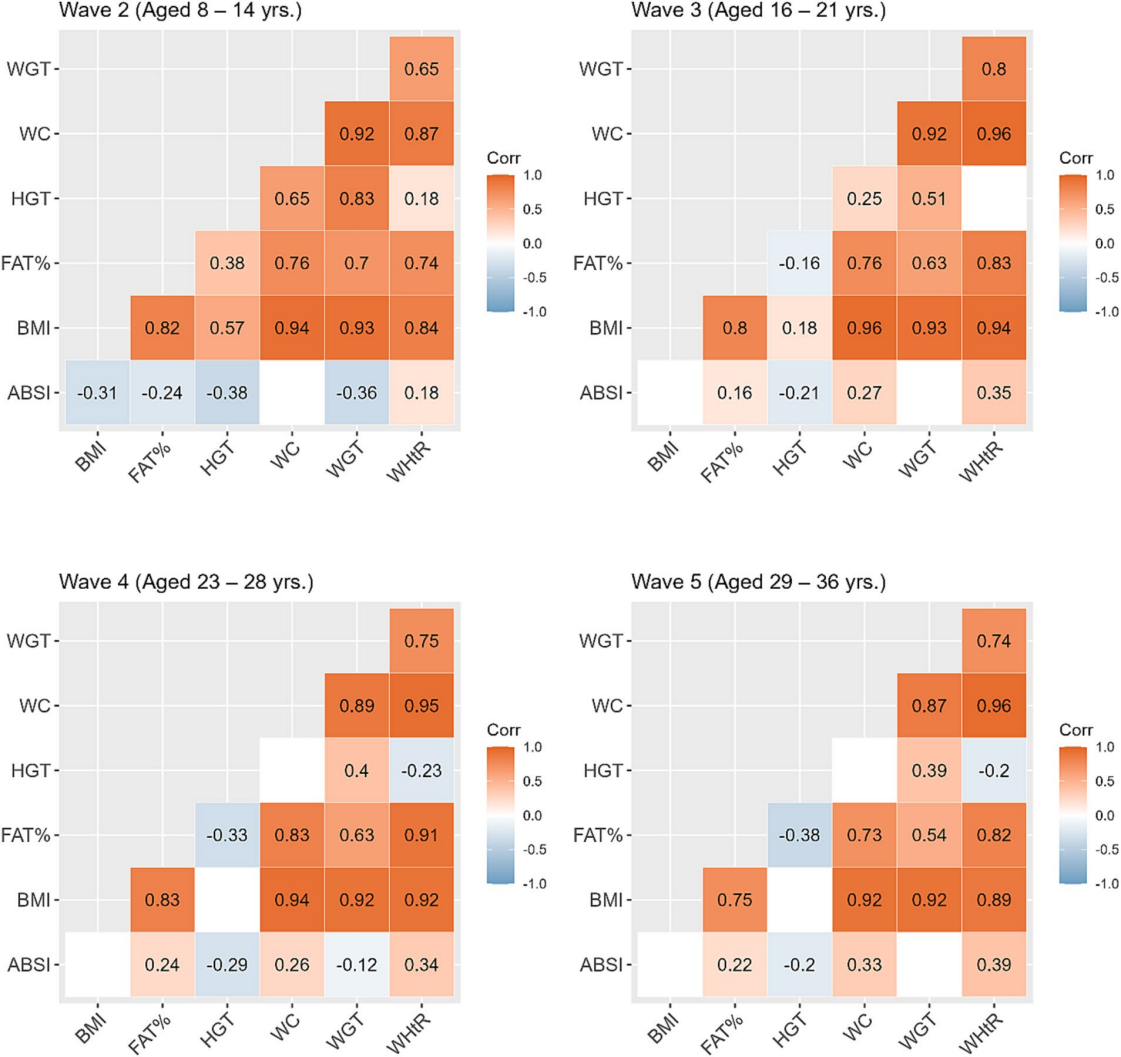
Correlations between the anthropometric measures within each study wave. Blank cells represent non-significant correlations. ABSI, a body shape index; BMI, body mass index; FATP, fat percentage; HGT, height; WC, waist circumference; WGT, weight; WHtR, waist/height ratio.

Table 2 shows the mean and standard deviation (SD) for anthropometric measurements across the study waves. Compared to their non-SGA counterparts, individuals born SGA showed lower mean values for all anthropometric measures (except for WHtR) across all waves. In Figure 2, we present correlations between the anthropometric measures within each wave. Overall, BMI displayed the strongest positive correlations with other anthropometric measures (excluding height, *r* = 0.75 to 0.96). Additionally, WC exhibited consistently strong positive correlations with weight across all waves (*r* = 0.87 to 0.92). The positive correlation between height and weight was most pronounced during Wave 2 (*r* = 0.83), and then gradually diminished over the subsequent waves (*r* = 0.51, 0.40, 0.39 waves 3, 4, and 5 respectively). The ABSI exhibited a small negative correlation with BMI during Wave 2 (*r* = −0.31), whereas for subsequent waves BMI and ABSI were not significantly correlated.

**Table 2:** Mean (SD) anthropometric measures from childhood to adult.

	Wave 2 (Aged 8–14 yrs.)		Wave 3 (Aged 16–21 yrs.)		Wave 4 (Aged 23–28 yrs.)		Wave 5 (Aged 29–36 yrs.)	
	*n*	Mean (SD)	*n*	Mean (SD)	*n*	Mean (SD)	*n*	Mean (SD)
Among all								
ABSI	537	0.082 (0.004)	415	0.080 (0.004)	394	0.082 (0.005)	355	0.083 (0.006)
BMI	569	17.0 (3.5)	422	21.6 (5.7)	404	23.7 (6.4)	359	25.3 (6.7)
Fat %	509	21.3 (9.4)	397	20.7 (12.1)	360	24.8 (12.1)	306	27.6 (10.0)
Height (cm)	570	143.8 (10.6)	423	167.8 (8.7)	405	167.9 (8.8)	359	167.5 (8.9)
Waist circ. (cm)	538	64.6 (9.6)	415	79.3 (14.8)	395	86.6 (15.7)	355	91.8 (17.1)
Weight (kg)	570	35.9 (11.8)	422	61.1 (19.2)	404	67.2 (20.1)	359	71.2 (20.4)
Waist/height ratio	538	0.4 (0.1)	415	0.5 (0.1)	395	0.5 (0.1)	355	0.5 (0.1)
Among SGA								
ABSI	107	0.083 (0.004)	80	0.080 (0.004)	74	0.083 (0.005)	71	0.084 (0.005)
BMI	114	15.9 (2.7)	83	20.0 (4.7)	75	21.8 (5.6)	71	23.5 (5.5)
Fat %	103	18.4 (8.2)	75	17.0 (10.3)	68	22.5 (11.4)	57	25.0 (10.1)
Height (cm)	115	142.8 (10.8)	83	164.5 (8.0)	76	164.7 (8.0)	71	164.4 (8.6)
Waist circ. (cm)	108	62.2 (7.8)	80	75.5 (12.7)	75	82.2 (13.1)	71	87.7 (13.7)
Weight (kg)	115	33.0 (9.8)	83	54.3 (13.7)	75	59.2 (15.3)	71	63.5 (15.0)
Waist/height ratio	108	0.4 (0.0)	80	0.5 (0.1)	75	0.5 (0.1)	71	0.5 (0.1)
Among non-SGA								
ABSI	430	0.082 (0.004)	335	0.080 (0.004)	320	0.082 (0.004)	282	0.083 (0.004)
BMI	455	17.2 (3.6)	339	21.9 (5.9)	329	24.2 (6.5)	288	25.8 (6.9)
Fat %	406	22.0 (9.5)	322	21.5 (12.3)	292	25.3 (12.2)	249	28.2 (9.9)
Height (cm)	455	144.1 (10.5)	340	168.6 (8.6)	329	168.7 (8.8)	288	168.2 (8.8)
Waist circ. (cm)	430	65.2 (9.9)	335	80.2 (15.2)	320	87.6 (16.1)	284	92.8 (17.7)
Weight (kg)	455	36.6 (12.2)	339	62.8 (19.9)	329	69.0 (20.7)	288	73.1 (21.1)
Waist/height ratio	430	0.5 (0.1)	335	0.5 (0.1)	320	0.5 (0.1)	284	0.6 (0.1)

Results from linear regression models examining factors associated with anthropometric z-scores can be found in Table 3. In summary, after controlling for sex, age, and residential location, individuals born SGA exhibited consistent reductions in anthropometric z-scores throughout the study waves compared to non-SGA individuals, with standardised coefficients ranging from −0.50 to −0.25. The only exception was WHtR, which showed no significant differences between SGA and non-SGA individuals in Waves 4 and 5. On the other hand, participants residing in urban environments were significantly larger for all anthropometric measurements within Waves 2 to 4 with coefficients ranging from 0.26 to 0.65. At Wave 5, this pattern only persisted for BMI and weight. The most substantial differences between genders were observed in FAT% with females consistently exhibiting higher values across all study waves (coefficients ranged from 1.06 to 1.19). Similarly, females also demonstrated higher WHtR values in Waves 3, 4, and 5 (coefficients ranged from 0.37 to 0.58) and weight (coefficients ranged from −0.42 to −0.33) in Waves 3, 4, and 5. Individuals who reported smoking consistently displayed reduced anthropometric measurements in Waves 3, 4, and 5, with the most prominent differences observed in Wave 5.

**Table 3:** Cross-sectional analyses: associations between anthropometric z-scores and potential correlates within each study wave.

	ABSI	BMI	Fat %	Height	Waist circ.	Weight	Waist/height ratio
	Coefficient (95% CI)	Coefficient (95% CI)	Coefficient (95% CI)	Coefficient (95% CI)	Coefficient (95% CI)	Coefficient (95% CI)	Coefficient (95% CI)
Wave 2 (Aged 8–14 yrs.)							
SGA	0.26 (0.06, 0.46)	**−0.42 (−0.61, −0.23)**	**−0.42 (−0.60, −0.24)**	−0.25 (−0.40, −0.10)	**−0.36 (−0.55, −0.17)**	**−0.38 (−0.55, −0.21)**	**−0.32 (−0.52, −0.11)**
Female	**−0.30 (−0.46, −0.14)**	0.22 (0.07, 0.37)	**1.06 (0.91, 1.20)**	0.22 (0.10, 0.34)	*0.13 (−0.02, 0.29)*	0.24 (0.10, 0.38)	*0.04 (−0.13, 0.21)*
Urban	**−0.54 (−0.73, −0.35)**	**0.64 (0.46, 0.81)**	**0.36 (0.18, 0.53)**	**0.50 (0.36, 0.64)**	**0.56 (0.38, 0.74)**	**0.65 (0.49, 0.81)**	**0.39 (0.19, 0.59)**
Age	−0.20 (−0.27, −0.13)	0.22 (0.15, 0.28)	0.18 (0.12, 0.24)	**0.58 (0.53, 0.63)**	0.28 (0.21, 0.35)	**0.40 (0.34, 0.46)**	*−0.01 (−0.08, 0.07)*
Wave 3 (Aged 16–21 yrs.)							
SGA	*0.06 (−0.19, 0.31)*	**−0.37 (−0.62, −0.13)**	**−0.42 (−0.64, −0.20)**	**−0.37 (−0.56, −0.19)**	**−0.38 (−0.62, −0.14)**	**−0.45 (−0.68, −0.22)**	−0.28 (−0.53, −0.04)
Female	**0.53 (0.33, 0.73)**	*0.05 (−0.14, 0.24)*	**1.07 (0.90, 1.24)**	**−1.27 (−1.41, −1.12)**	*0.00 (−0.19, 0.20)*	**−0.38 (−0.56, −0.20)**	**0.37 (0.18, 0.57)**
Urban	*−0.14 (−0.40, 0.12)*	**0.56 (0.31, 0.81)**	**0.38 (0.16, 0.61)**	0.26 (0.06, 0.45)	**0.51 (0.26, 0.77)**	**0.61 (0.37, 0.85)**	**0.44 (0.19, 0.70)**
Age	*0.04 (−0.05, 0.13)*	*0.03 (−0.05, 0.12)*	*−0.01 (−0.09, 0.07)*	0.01 (−0.05, 0.08)	*0.05 (−0.04, 0.14)*	*0.04 (−0.05, 0.12)*	*0.05 (−0.04, 0.14)*
Smoking	*0.17 (−0.05, 0.38)*	−0.28 (−0.49, −0.08)	−0.20 (−0.38, −0.01)	0.00 (−0.16, 0.15)	−0.22 (−0.43, −0.01)	−0.26 (−0.45, −0.06)	−0.22 (−0.43, −0.01)
Alcohol	*0.05 (−0.18, 0.27)*	**0.34 (0.12, 0.55)**	0.26 (0.07, 0.45)	0.21 (0.04, 0.37)	**0.35 (0.13, 0.56)**	**0.36 (0.16, 0.56)**	**0.30 (0.08, 0.52)**
Wave 4 (Aged 23–28 yrs.)							
SGA	**0.37 (0.12, 0.62)**	**−0.41 (−0.67, −0.14)**	**−0.30 (−0.54, −0.06)**	**−0.38 (−0.57, −0.19)**	**−0.37 (−0.63, −0.11)**	**−0.50 (−0.75, −0.24)**	*−0.26 (−0.51, 0.00)*
Female	**0.69 (0.48, 0.90)**	*0.19 (−0.03, 0.40)*	**1.13 (0.93, 1.33)**	**−1.35 (−1.51, −1.20)**	*0.19 (−0.03, 0.40)*	**−0.33 (−0.54, −0.13)**	**0.58 (0.37, 0.80)**
Urban	*0.00 (−0.24, 0.25)*	**0.49 (0.23, 0.74)**	0.29 (0.05, 0.53)	0.28 (0.09, 0.46)	**0.49 (0.24, 0.75)**	**0.56 (0.31, 0.80)**	**0.40 (0.14, 0.65)**
Age	*−0.04 (−0.12, 0.05)*	*0.08 (−0.01, 0.17)*	*0.06 (−0.02, 0.15)*	*0.00 (−0.06, 0.07)*	*0.07 (−0.02, 0.16)*	*0.07 (−0.02, 0.16)*	*0.07 (−0.02, 0.16)*
Smoking	*0.03 (−0.19, 0.25)*	−0.29 (−0.52, −0.06)	−0.26 (−0.47, −0.05)	*−0.04 (−0.20, 0.13)*	−0.28 (−0.51, −0.05)	*−0.29 (−0.51, −0.07)*	−0.26 (−0.49, −0.04)
Alcohol	−0.27 (−0.50, −0.05)	*0.15 (−0.08, 0.38)*	*0.12 (−0.09, 0.33)*	*0.15 (−0.02, 0.32)*	*0.12 (−0.12, 0.35)*	*0.16 (−0.07, 0.38)*	*0.08 (−0.15, 0.31)*
Wave 5 (Aged 29–36 yrs.)							
SGA	*0.17 (−0.09, 0.43)*	**−0.33 (−0.58, −0.08)**	−0.29 (−0.53, −0.06)	**−0.48 (−0.66, −0.30)**	**−0.31 (−0.56, −0.05)**	**−0.49 (−0.73, −0.25)**	*−0.16 (−0.41, 0.10)*
Female	**0.52 (0.31, 0.74)**	*0.14 (−0.07, 0.34)*	**1.19 (1.01, 1.38)**	**−1.44 (−1.58, −1.29)**	*0.08 (−0.13, 0.30)*	**−0.42 (−0.62, −0.22)**	**0.48 (0.27, 0.69)**
Urban	*−0.19 (−0.49, 0.10)*	**0.34 (0.05, 0.63)**	*0.15 (−0.11, 0.41)*	0.22 (0.02, 0.43)	*0.27 (−0.03, 0.56)*	**0.41 (0.13, 0.69)**	*0.20 (−0.09, 0.49)*
Age	*0.01 (−0.06, 0.09)*	*0.03 (−0.04, 0.10)*	*0.01 (−0.05, 0.08)*	*0.00 (−0.05, 0.05)*	*0.04 (−0.03, 0.11)*	*0.03 (−0.03, 0.10)*	*0.04 (−0.03, 0.11)*
Smoking	*0.08 (−0.16, 0.32)*	**−0.64 (−0.87, −0.41)**	**−0.39 (−0.60, −0.18)**	*0.04 (−0.13, 0.20)*	**−0.56 (−0.80, −0.32)**	**−0.60 (−0.82, −0.37)**	**−0.55 (−0.78, −0.32)**
Alcohol	*−0.13 (−0.36, 0.10)*	0.25 (0.03, 0.47)	0.21 (0.01, 0.41)	*0.09 (−0.06, 0.25)*	*0.22 (−0.01, 0.44)*	0.27 (0.06, 0.48)	*0.18 (−0.04, 0.40)*

All potential correlates are entered into the models simultaneously. Wave 2 models included sex, age, SGA status, and residential location. Wave 3, 4, and 5 models included sex, age, SGA status, residential location, smoking, and alcohol use. To assist in observing stronger associations, figures in **bold** represent significant associations where the coefficient ≥ 0.30 (absolute). Figures italicised represent non-significant associations.

For ABSI scores, individuals born SGA displayed significantly higher scores in Waves 2 and 4 (coefficients = 0.26 and 0.37, respectively), whereas SGA was not associated with ABSI in other study waves. Females had significantly lower ABSI scores during Wave 2, however, this shifted over time with females exhibiting significantly higher ABSI scores in later waves. No other factors were found to be associated with ABSI scores, except for urban residents in Wave 2, who had significantly lower ABSI scores compared to their remote counterparts.

In all models presented in Table 3, we conducted further investigations to examine the influence of an interaction term involving SGA and residential location. Results showed statistically significant interaction terms only during Wave 4 (results not shown). In urban settings, non-SGA individuals exhibited significantly larger values across anthropometric measures compared to their SGA counterparts.

### Longitudinal analyses

Based on the linear mixed models, the post-hoc contrasts between SGA and non-SGA are shown in Table 4. When estimating the marginal means for anthropometric measures (z-scores) at the ages of 11, 18, 25, and 32 years, contrasts showed that in the urban regions SGA individuals were consistently smaller than their non-SGA peers at 11, 18, and 25 years for BMI (differences = −0.35, −0.57, −0.60, respectively), FAT% (differences = −0.47, −0.62, −0.54, respectively), height (differences = −0.20, −0.43, −0.35, respectively), WC (differences = −0.37, −0.55, −0.66, respectively), weight (differences = −0.31, −0.67, −0.67, respectively), and WHtR (differences = −0.30, −0.44, −0.59, respectively). Whereas in remote communities, similar but smaller differences were observed between SGA and non-SGA at 11, 18, 25 and 32 years for BMI (differences = −0.21, −0.19, −0.33, −0.39, respectively), FAT% (differences = −0.26, −0.24, −0.25, −0.27, respectively), height (differences = −0.16, −0.21, −0.22, −0.24, respectively), WC (differences at 11, 25, and 32 years = −0.18, −0.21, −0.28, respectively), and weight (differences = −0.19, −0.25, −0.37, −0.42, respectively).

**Table 4:** Longitudinal analyses: differences in anthropometric z-score marginal means between SGA and non-SGA (reference) estimated at 11, 18, 25, and 32 years of age, stratified by residential location.

*Stratified by*	ABSI	BMI	Fat %	Height	Waist circ.	Weight	Waist/height ratio
	Difference (95%CI)	Difference (95%CI)	Difference (95%CI)	Difference (95%CI)	Difference (95%CI)	Difference (95%CI)	Difference (95%CI)
Age 11							
Remote	0.14 (−0.05, 0.33)	**−0.21 (−0.34, −0.07)**	**−0.26 (−0.44, −0.08)**	−0.16 (−0.29, −0.03)	−0.18 (−0.31, −0.04)	−0.19 (−0.30, −0.07)	−0.16 (−0.31, −0.01)
Urban	0.42 (0.00, 0.85)	**−0.35 (−0.57, −0.14)**	**−0.47 (−0.82, −0.13)**	**−0.20 (−0.42, 0.02)**	**−0.37 (−0.63, −0.11)**	**−0.31 (−0.50, −0.13)**	**−0.31 (−0.59, −0.02)**
Age 18							
Remote	**0.26 (0.04, 0.48)**	−0.19 (−0.36, −0.02)	**−0.24 (−0.43, −0.05)**	**−0.21 (−0.33, −0.10)**	*−0.12 (−0.28, 0.04)*	**−0.25 (−0.40, −0.10)**	*−0.05 (−0.23, 0.13)*
Urban	−0.06 (−0.50, 0.38)	**−0.57 (−0.83, −0.31)**	**−0.62 (−0.96, −0.27)**	**−0.43 (−0.62, −0.24)**	**−0.55 (−0.83, −0.28)**	**−0.67 (−0.90, −0.45)**	−**0.44 (−0.75, −0.12)**
Age 25							
Remote	**0.34 (0.10, 0.59)**	**−0.33 (−0.54, −0.12)**	**−0.25 (−0.45, −0.05)**	**−0.22 (−0.33, −0.11)**	**−0.21 (−0.41, −0.02)**	**−0.37 (−0.56, −0.18)**	*−0.14 (−0.37, 0.09)*
Urban	−0.04 (−0.54, 0.46)	**−0.60 (−0.94, −0.26)**	**−0.54 (−0.93, −0.14)**	**−0.35 (−0.56, −0.15)**	**−0.66 (−1.01, −0.32)**	**−0.67 (−0.98, −0.37)**	**−0.59 (−1.00, −0.19)**
Age 32							
Remote	0.28 (0.00, 0.56)	**−0.39 (−0.65, −0.13)**	**−0.27 (−0.50, −0.05)**	**−0.24 (−0.36, −0.11)**	**−0.28 (−0.52, −0.04)**	**−0.42 (−0.66, −0.19)**	*−0.20 (−0.48, 0.08)*
Urban	−0.18 (−0.91, 0.55)	*−0.31 (−0.80, 0.19)*	*−0.06 (−0.62, 0.49)*	*−0.28 (−0.59, 0.03)*	*−0.43 (−0.94, 0.08)*	*−0.38 (−0.82, 0.05)*	*−0.38 (−0.98, 0.22)*

These are post-hoc contrasts from the linear mixed models with interaction terms between SGA and residential location. Models included sex, age, SGA status, and residential location. To assist in observing stronger associations, figures in **bold** represent significant differences ≥ 0.20 (absolute). Figures italicised represent non-significant differences.

For the ABSI, in remote communities the SGA individuals had significantly higher scores at the ages of 18 years (difference = 0.26) and 25 years (difference = 0.34), whereas there was no difference in the ABSI between SGA and non-SGA in urban regions. For the WHtR, in urban regions SGA individuals were significantly smaller at the ages of 11 years (difference = −0.31), 18 years (difference = −0.44), and 25 years (difference = −0.59), but not at 32 years. Whereas, in remote communities WHtR was only smaller among SGA at 11 years (difference = −0.16).

**Figure 3:**
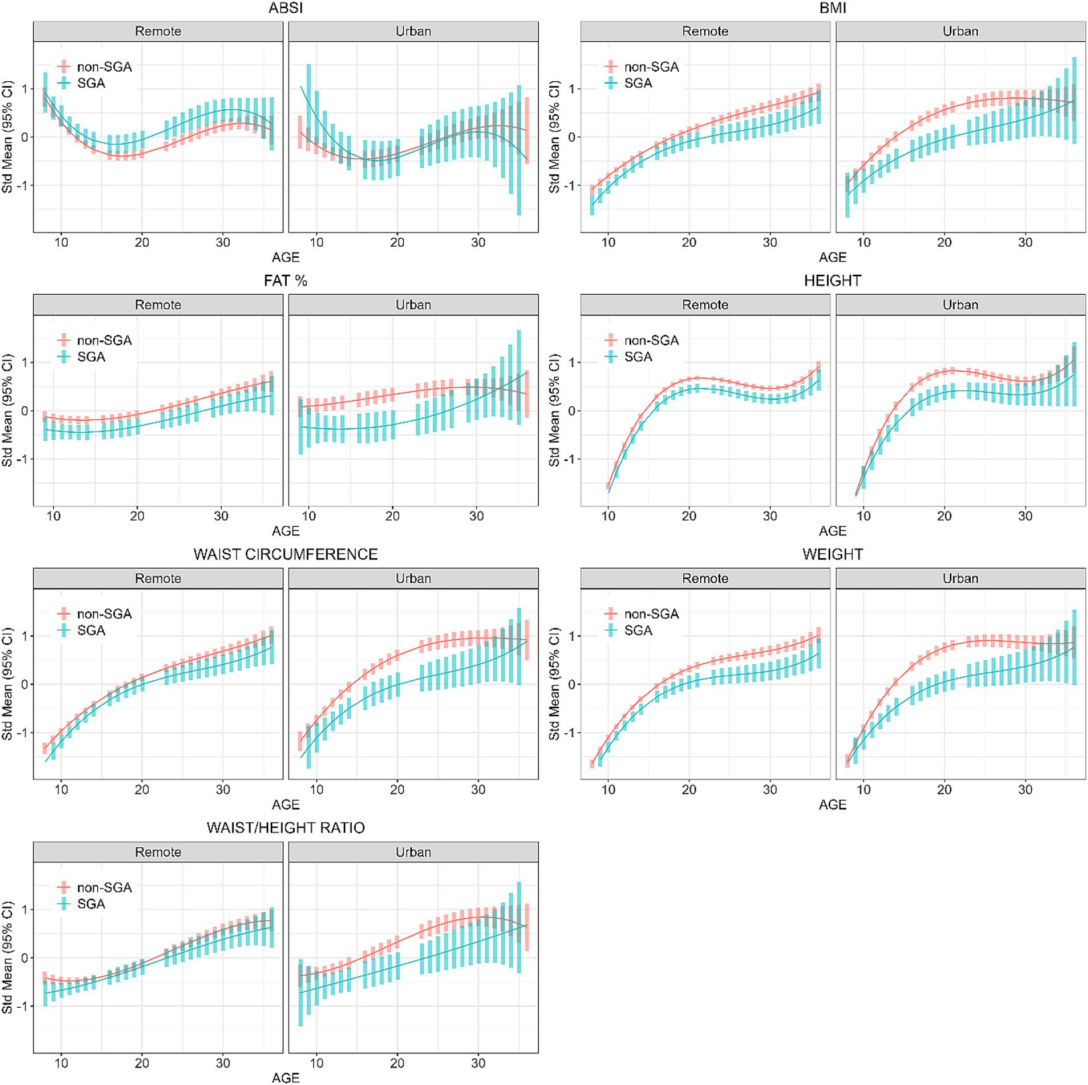
Longitudinal analyses: estimated anthropometric z-scores for ages across all waves, stratified by SGA status and Location. Models included sex, age, SGA status, and residential location.

Interpretation of Figure 3 shows that in remote communities the anthropometric trajectories followed a similar pattern for both SGA and non-SGA individuals, with significant differences for all measures except for WC and WHtR. In contrast, differences between SGA and non-SGA individuals in urban environments were most pronounced during the late-teens through mid-twenties, particularly for BMI, FAT%, WC, and weight. However, it’s worth noting that these differences between SGA and non-SGA individuals in urban environments diminished towards 30 years of age. These results further confirm the interactions between SGA and residential location that were initially identified in Wave 4 cross-sectional analyses.

**Figure 4:**
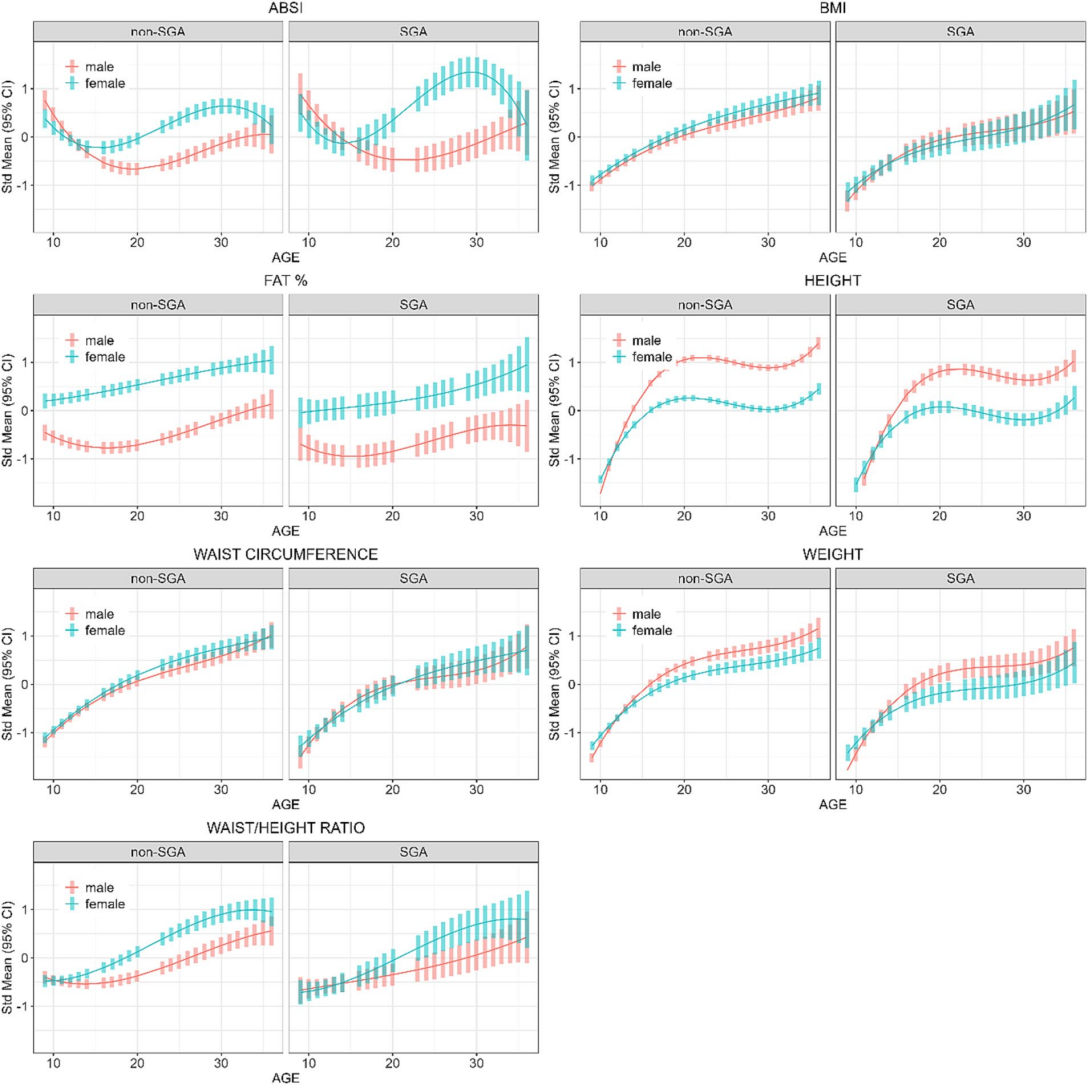
Longitudinal analyses within remote communities: estimated anthropometric z-scores for ages across all waves, stratified by sex and SGA status. Models included sex, age, SGA status.

Further analyses were restricted to those residing in remote communities to assess the differences in anthropometric measures across genders and SGA status. Table 5 shows the post-hoc contrasts between females and males (reference) estimated at the ages 11, 18, 25, and 32 years, stratified by SGA status. At age 18 years females had significantly higher ABSI scores than males, and the magnitude of these differences were similar in the SGA (gender difference = 0.50) and non-SGA groups (gender difference = 0.48). Then at ages 25 and 32 years the magnitude of the difference in ABSI score between females and males was almost two-fold in the SGA group (at 25 and 32 years: gender difference = 1.47 and 1.19 respectively) than in the non-SGA group (at 25 and 32 years: gender difference = 0.85 and 0.65). Females also had significantly higher FAT% and WHtR but the differences were relatively similar in the SGA and non-SGA groups. Figure 4 shows the estimated marginal means for males and females in remote communities, stratified by SGA status.

**Table 5:** Longitudinal analyses: within remote communities, differences in anthropometric z-score marginal means between female and male (reference) estimated at 11, 18, 25, and 32 years of age, stratified by SGA status.

*Stratified by*	ABSI	BMI	Fat %	Height	Waist circ.	Weight	Waist/height ratio
	Difference (95%CI)	Difference (95%CI)	Difference (95%CI)	Difference (95%CI)	Difference (95%CI)	Difference (95%CI)	Difference (95%CI)
Age 11							
Non-SGA	−0.18 (−0.35, −0.01)	*0.10 (−0.01, 0.21)*	**0.85 (0.69, 1.02)**	0.11 (0.01, 0.21)	*0.06 (−0.05, 0.17)*	*0.08 (−0.01, 0.18)*	*0.04 (−0.08, 0.17)*
SGA	**−0.36 (−0.69, −0.03)**	*0.09 (−0.12, 0.31)*	**0.83 (0.51, 1.16)**	**0.23 (0.04, 0.43)**	*0.05 (−0.16, 0.27)*	*0.13 (−0.06, 0.31)*	*−0.05 (−0.29, 0.19)*
Age 18							
Non-SGA	**0.48 (0.30, 0.66)**	*0.11 (−0.04, 0.25)*	**1.22 (1.05, 1.39)**	**−0.70 (−0.80, −0.61)**	*0.10 (−0.04, 0.24)*	**−0.24 (−0.36, −0.11)**	**0.42 (0.26, 0.58)**
SGA	**0.50 (0.13, 0.86)**	*−0.09 (−0.38, 0.19)*	**1.04 (0.70, 1.38)**	**−0.61 (−0.80, −0.43)**	*−0.08 (−0.36, 0.19)*	**−0.34 (−0.59, −0.10)**	*0.19 (−0.12, 0.50)*
Age 25							
Non-SGA	**0.85 (0.64, 1.06)**	*0.18 (−0.02, 0.37)*	**1.20 (1.01, 1.39)**	**−0.88 (−0.97, −0.79)**	*0.19 (0.00, 0.37)*	**−0.31 (−0.47, −0.14)**	**0.65 (0.44, 0.85)**
SGA	**1.47 (1.07, 1.87)**	*−0.09 (−0.46, 0.28)*	**0.93 (0.56, 1.30)**	**−0.85 (−1.03, −0.68)**	*0.12 (−0.23, 0.47)*	**−0.43 (−0.75, −0.12)**	**0.54 (0.14, 0.93)**
Age 32							
Non-SGA	**0.65 (0.42, 0.89)**	*0.18 (−0.07, 0.42)*	**1.01 (0.80, 1.22)**	**−0.86 (−0.96, −0.76)**	*0.13 (−0.10, 0.36)*	**−0.34 (−0.54, −0.13)**	**0.60 (0.34, 0.86)**
SGA	**1.19 (0.74, 1.63)**	*0.03 (−0.43, 0.50)*	**0.98 (0.57, 1.39)**	**−0.80 (−0.98, −0.61)**	*0.17 (−0.27, 0.60)*	*−0.36 (−0.75, 0.04)*	**0.60 (0.11, 1.10)**

These are post-hoc contrasts from the linear mixed models with interaction terms between SGA and sex. Models included sex, age, SGA status. To assist in observing stronger associations, figures in **bold** represent significant differences ≥ 0.20 (absolute). Figures italicised represent non-significant differences.

### Sensitivity analyses

For the linear mixed models, we included tobacco smoking and alcohol use and only analysed data from Wave 3 onwards. The estimated marginal means at the ages of 18, 25, and 32 years can be found in Supplementary Table S1. Given that study participation was non-continuous, we limited the cross-sectional and longitudinal analyses to those who participated in all waves (*n* = 283) and results were similar to the those yielded in the main analyses, however SGA individuals were not significantly smaller across all anthropometric measures in Waves 4 and 5 (Supplementary Tables S2, S3). Within each wave, we checked for differences in anthropometric measures among those who continued to participate in the subsequent wave versus those who did not participate in the next wave. Results showed no differences in anthropometric measures for the transition of Wave 2 to Wave 3; for Wave 3 to Wave 4 those who did not continue to Wave 4 had significantly higher BMI, weight and WC; for Wave 4 to Wave 5 those who did not continue to Wave 5 had significantly higher WC and weight (Supplementary Table S4).

To compare the ABC study participant ABSI scores to those originally derived from the NHANES, we downloaded the data from the original authors (18) and plotted the ABSI scores for the ABC and NHANES cohorts across the age groups stratified by gender (Supplementary Figure S1). It was observed that the ABC study participants had significantly higher ABSI scores than the NHANES cohort, with more prominent differences among older females.

## Discussion

We investigated the longitudinal changes in anthropometric parameters among Indigenous Australians who were born SGA compared to those born non-SGA. Our results consistently demonstrate lower anthropometric measurements in the SGA individuals from childhood through young adult, indicating that through their life course those born SGA are smaller than those born non-SGA. Although current literature on SGA and body composition later in life among Indigenous Australians is limited, our findings align with other studies (from Sweden, Netherlands, Japan, Greece, and a meta-analysis) reporting that individuals born SGA tend to be smaller during childhood (23–27).

Within each study wave most anthropometric measures were positively correlated (except for correlations with ABSI). Hence, the magnitude of the differences between SGA and non-SGA individuals were similar across different anthropometric measures, except for WHtR yielding no difference during the two latest waves. However, the disparity in anthropometric measures between SGA and non-SGA individuals was more evident in urban communities where non-SGA are larger than their SGA counterparts, in addition to being larger than both SGA and non-SGA in remote communities. Indigenous Australians nutritional intake has transitioned since colonisation from a traditional, varied and nutrient-dense diet, high in fibre and low in fat and refined carbohydrates, to an energy-dense westernised diet, high in fat and refined sugars (28). The geographic differential observed indicates the strong influence that residential environments have on growth and body composition, which could be driven by lifestyle factors such as dietary options available, food insecurity, and financial stress in the remote Indigenous communities (15, 17, 29), and/or people in urban areas having greater access to supermarkets and fast food outlets and a high proportion of energy-dense, nutrient-poor diet (28).

As expected, other publications analysing the anthropometric data from the ABC study have also reported this geographical divide with individuals in urban regions being larger compared to their counterparts in remote areas (15, 17, 30–32). This geographic differential has also been highlighted in other measures of health within the ABC study. For instance, Indigenous children residing in remote areas exhibit lower markers of lung function (33), haemoglobin levels, total cholesterol levels and systolic blood pressure (30), while Indigenous young adults residing in remote areas exhibit longer cognitive reaction times (34), lower maximal grip strength (32), lower iodine levels (35), and Indigenous females in remote areas exhibit adverse cardio-metabolic profiles (31).

BMI is a widely adopted metric for evaluating overall obesity by considering an individual’s weight and height. It has limitations as it does not consider factors such as body shape or the distribution of muscle mass versus fat mass (36). In our study, BMI was observed to be lower among SGA individuals in both remote and urban regions with the difference in urban regions being far more pronounced. This is consistent with other studies reporting that those born SGA have a lower BMI later in life (26, 37). Two Australian studies analysing data from a remote Indigenous community in the Northern Territory found BMI to be inversely associated with all-cause mortality after follow-up at 9 years (38) and 18 years (39), however, increased WC was associated with increased risk of all-cause mortality (39). In the context of our study, these findings would suggest conflicting mortality risks for SGA individuals given that they showed significantly lower BMI and WC compared to those born non-SGA. It is important to note however, other large studies of non-Indigenous populations have reported a J-shape relationship between BMI and mortality showing that both lower and higher BMI is associated with increased risk of mortality (40, 41).

ABSI, on the other hand, incorporates WC as a key component in its calculation, and therefore intended to provide a better measure of central adiposity (18). In support of ABSI being independent of BMI our results showed no significant correlation between ABSI and BMI in Waves 3, 4, and 5. The ABSI and WHtR were the only two anthropometric measures that were inconsistent across the ages in terms of the contrasts between SGA and non-SGA individuals. In remote communities ABSI scores were significantly elevated among those born SGA compared to their non-SGA counterparts, and in urban regions there was no difference in ABSI scores between SGA and non-SGA despite the urban non-SGA individuals being the largest group across most anthropometric measures. This difference between SGA and non-SGA in ABSI scores in remote communities first appeared from adolescence, indicating an early-life predisposition to central adiposity among SGA individuals in remote communities. Earlier research among the ABC study participants at childhood showed that many had lower BMI with higher waist measurements (42).

Hence, although SGA individuals in remote communities are smaller in size, their higher ABSI scores indicate greater central adiposity. This interesting finding highlights the potential complex interplay between possible early catch-up growth among those born SGA, lifestyle factors later in life, and the nutritional challenges prevalent in remote communities (29, 43). Rapid catch-up growth among SGA infants is a risk factor for being overweight/obese at 2–5 years of age (9) and is associated with increased fat mass at 9 years of age (37), and fat mass with central adiposity at 32 years of age (44). Lower birth weight combined with faster growth in the first 5 years has also been associated with increased hepatic fat in early childhood (45). Given that the first follow-up of the ABC study participants was at 8 years of age (Wave 2), we were unable to determine if any of our results, particularly higher ABSI scores (e.g., higher central adiposity) among those born SGA, are associated with catch-up growth during infancy. However, based on the assumption that Indigenous Australians in remote communities most likely experience similar lifestyle and nutritional challenges, it is therefore possible that individuals born SGA and residing in remote communities may exhibit a propensity for central adiposity, potentially mediated by the biological processes of catch-up growth and lifestyle factors. Consistent with this view, a study of 128 Australian Indigenous children found that children who experienced rapid weight gain in the first 12 months of life were 2.7 times more likely to be overweight at 9 years. Although the analyses were not stratified by gender, female children were 2.4 times more likely to be overweight at 9 years compared to male children (46).

Our cross-sectional findings (in models including SGA status) showed that, although females weighed less, they had higher FAT% with a larger WHtR and ABSI scores and this was more evident in remote communities. In analyses stratified by SGA status, the gender disparity in ABSI scores among SGA individuals was nearly twofold greater than observed in the non-SGA group. This observation implies that in remote communities, Indigenous females born SGA tend to accrue a higher degree of central adiposity than SGA males by late adolescence. In support of this, other research conducted within the ABC study showed that Indigenous females residing in remote communities exhibit the highest predicted likelihood of having an adverse cardio-metabolic profile, a trend that was consistent across all levels of BMI. Furthermore, this pattern was not observed among their male counterparts (31).

To the best of our knowledge, there are no prior studies specifically investigating ABSI within Indigenous communities, making direct comparisons with existing research challenging. However, it is worth noting a recent study conducted on a sample of Australian adults (*n* = 4,056) reported a dose–response association with mortality across ABSI quartiles (47). In an Italian study of overweight and obese children aged 2–18 years, higher ABSI scores were associated with higher cardio-metabolic risk markers (48). Other studies have also reported that the ABSI performs well in predicting all-cause mortality (18, 49–51), hypertension (50), arterial stiffness in patients with type 2 diabetes (52), cardiovascular disease risk (53), and cancer (54).

Although research on SGA and body composition among Indigenous Australians is limited, studies among minority groups in low- and middle-income countries have reported positive associations between birth weight and body composition later in life with differences across genders. For example, in the Inuit population of Greenland (aged 18–61 years), birth weight was positively associated with BMI, waist circumference, fat mass index, fat-free mass index, and subcutaneous adipose tissue with weaker associations among females compared to males (55). In South Western Townships (Soweto), South Africa, a study of young adults (aged 22 years) reported that birth weight was positively associated with fat mass in males only, while relative weight gain in early life was associated with visceral adipose tissue in females (56). Another study in South Africa (infants aged 24 months) reported positive associations between weight gain and fat mass, fat-free mass, fat mass index, and fat-free mass index with no difference between genders (57). In Mexico, a study of Maya children (mean age 7.5 years) indicated that birth weight was positively associated with fat-free mass index and fat mass index among males only (58). A study of Brazilian adults (aged 35–74 years) found that low birth weight predicted higher levels of truncal fat in females but not in males (59, 60).

### Limitations

Several limitations must be acknowledged in our study. Firstly, the children were first followed-up at 8 years of age, therefore early catch-up growth could not be analysed. Secondly, our study classified individuals into either remote or urban residential categories; however, we lacked information on the precise duration spent in each location, which complicates the ability to draw definitive conclusions regarding the association between residential location and growth patterns. Thirdly, although convenient, inexpensive and easy to use, Bioelectrical Impedance Analysis (BIA) underestimates body fat in the severely obese compared to DEXA (61). Lastly, a large portion of the study population resided in remote areas, limiting our ability to explore stratified analyses within the urban context.

## Conclusion

The findings of our study showed that Indigenous individuals born SGA are smaller anthropometrically later in life compared to their non-SGA counterparts, and this disparity was more pronounced in urban regions. However, SGA individuals in remote communities had higher ABSI scores than non-SGA individuals, indicating that SGA individuals were more prone to accruing central adiposity. This result was driven by females in remote communities having higher ABSI and fat percentage than males, which was more evident among those born SGA. These findings highlight the complex relationships between early growth patterns, residential location, gender disparities, and central adiposity among Australian Indigenous born SGA.

## Data availability statement

The original contributions presented in the study are included in the article/Supplementary materials, further inquiries can be directed to the corresponding author: belinda.davison@menzies.edu.au.

## Ethics statement

The studies involving humans were approved by Human Research Ethics Committee of Northern Territory Health and Menzies School of Health Research (NT HREC). The studies were conducted in accordance with the local legislation and institutional requirements. Written informed consent for participation in this study was provided by the participants’ legal guardians/next of kin.

## Author contributions

CH: Data curation, Formal analysis, Methodology, Writing – original draft. BD: Conceptualization, Data curation, Funding acquisition, Investigation, Methodology, Project administration, Resources, Writing – review & editing. GS: Conceptualization, Data curation, Funding acquisition, Investigation, Methodology, Project administration, Resources, Writing – review & editing.
