# Supplementary material for: Small for gestational age and anthropometric body composition from early childhood to adulthood: the Aboriginal Birth Cohort study
Source: Front Public Health. 2024 Feb 21;12:1349040. doi: 10.3389/fpubh.2024.1349040 (PMC10915257; doi:10.3389/fpubh.2024.1349040)
Supplement: Supplementary file 1 [file Data_Sheet_1.docx]

**SUPPLEMENTAL TABLES AND FIGURES**

| **SUPPLEMENTAL TABLE S1:** Longitudinal analyses: Differences in anthropometric z-score marginal means between SGA and non-SGA (reference) estimated at 18, 25, and 32 years of age, stratified by residential location. These are post-hoc contrasts from the linear mixed models with interaction terms between SGA and residential location. These models are the same as those presented in Table 4 but with the inclusion of tobacco smoking and alcohol use. | | | | | | | |
| --- | --- | --- | --- | --- | --- | --- | --- |
| *Stratified* | **ABSI** | **BMI** | **Fat %** | **Height** | **Waist circ.** | **Weight** | **Waist/height ratio** |
| *by* | Difference (95%CI) | Difference (95%CI) | Difference (95%CI) | Difference (95%CI) | Difference (95%CI) | Difference (95%CI) | Difference (95%CI) |
| **Age 18** |  |  |  |  |  |  |  |
| Remote | 0.23 ( 0.00, 0.47) | **-0.26 (-0.47, -0.06)** | **-0.33 (-0.57, -0.09)** | **-0.26 (-0.35, -0.17)** | *-0.20 (-0.40, 0.01)* | **-0.32 (-0.50, -0.15)** | *-0.12 (-0.35, 0.11)* |
| Urban | **-0.41 (-0.91, 0.09)** | **-0.48 (-0.82, -0.13)** | **-0.54 (-0.97, -0.11)** | **-0.27 (-0.38, -0.15)** | **-0.58 (-0.95, -0.21)** | **-0.55 (-0.84, -0.26)** | **-0.49 (-0.91, -0.07)** |
| **Age 25** |  |  |  |  |  |  |  |
| Remote | **0.43 ( 0.18, 0.68)** | **-0.39 (-0.60, -0.17)** | **-0.34 (-0.55, -0.13)** | **-0.25 (-0.34, -0.16)** | **-0.24 (-0.44, -0.03)** | **-0.43 (-0.61, -0.24)** | *-0.15 (-0.38, 0.08)* |
| Urban | *-0.21 (-0.71, 0.29)* | **-0.42 (-0.76, -0.07)** | **-0.49 (-0.89, -0.09)** | **-0.24 (-0.36, -0.13)** | **-0.63 (-0.99, -0.28)** | **-0.48 (-0.76, -0.19)** | **-0.60 (-1.01, -0.19)** |
| **Age 32** |  |  |  |  |  |  |  |
| Remote | *0.21 (-0.09, 0.51)* | **-0.44 (-0.69, -0.18)** | *-0.21 (-0.42, 0.01)* | **-0.24 (-0.33, -0.14)** | **-0.34 (-0.59, -0.10)** | **-0.47 (-0.68, -0.26)** | *-0.27 (-0.55, 0.00)* |
| Urban | *-0.20 (-0.94, 0.55)* | *-0.22 (-0.72, 0.28)* | *-0.17 (-0.67, 0.33)* | **-0.23 (-0.36, -0.10)** | *-0.45 (-0.96, 0.07)* | *-0.34 (-0.74, 0.05)* | *-0.36 (-0.96, 0.24)* |
| Models included sex, age, SGA status, residential location, and tobacco smoking.  To assist in observing stronger associations, figures in **bold** represent significant differences ≥ 0.20 (absolute). Figures *italicised* represent non-significant differences. | | | | | | | |

| **SUPPLEMENTAL TABLE S2:** Cross-sectional analyses: Standardised regression coefficients for anthropometric measures within each wave among those who completed all study waves (n = 283). These models are the same as those presented in Table 3 but only among those who completed all study waves. | | | | | | | |
| --- | --- | --- | --- | --- | --- | --- | --- |
|  | **ABSI** | **BMI** | **Fat %** | **Height** | **Waist circ.** | **Weight** | **Waist/height ratio** |
|  | Coefficient (95% CI) | Coefficient (95% CI) | Coefficient (95% CI) | Coefficient (95% CI) | Coefficient (95% CI) | Coefficient (95% CI) | Coefficient (95% CI) |
| **Wave 2 (Aged 8 – 14 yrs.)** | |  |  |  |  |  |  |
| SGA | *0.15 (-0.15, 0.45)* | **-0.46 (-0.74, -0.18)** | **-0.38 (-0.65, -0.10)** | **-0.35 (-0.57, -0.13)** | **-0.45 (-0.74, -0.16)** | **-0.48 (-0.73, -0.22)** | **-0.36 (-0.68, -0.03)** |
| Female | **-0.35 (-0.58, -0.13)** | 0.25 ( 0.04, 0.47) | **1.05 ( 0.85, 1.25)** | 0.22 ( 0.05, 0.39) | *0.12 (-0.09, 0.34)* | 0.26 ( 0.07, 0.46) | *0.03 (-0.21, 0.27)* |
| Urban | **-0.65 (-1.01, -0.29)** | **0.76 ( 0.44, 1.08)** | **0.51 ( 0.18, 0.84)** | **0.54 ( 0.29, 0.80)** | **0.69 ( 0.34, 1.03)** | **0.76 ( 0.48, 1.05)** | **0.55 ( 0.16, 0.93)** |
| Age | -0.26 (-0.36, -0.16) | **0.30 ( 0.20, 0.39)** | 0.23 ( 0.14, 0.32) | **0.61 ( 0.53, 0.68)** | **0.35 ( 0.26, 0.45)** | **0.46 ( 0.38, 0.55)** | *0.03 (-0.07, 0.14)* |
| **Wave 3 (Aged 16 – 21 yrs.)** | |  |  |  |  |  |  |
| SGA | *0.13 (-0.19, 0.45)* | **-0.41 (-0.73, -0.09)** | **-0.51 (-0.79, -0.23)** | **-0.46 (-0.69, -0.22)** | **-0.41 (-0.73, -0.10)** | **-0.53 (-0.83, -0.23)** | *-0.27 (-0.58, 0.05)* |
| Female | **0.60 ( 0.36, 0.85)** | *0.09 (-0.15, 0.33)* | **1.08 ( 0.87, 1.29)** | **-1.28 (-1.46, -1.10)** | *0.07 (-0.18, 0.31)* | **-0.38 (-0.61, -0.16)** | **0.44 ( 0.19, 0.68)** |
| Urban | **-0.41 (-0.79, -0.03)** | **0.52 ( 0.14, 0.90)** | *0.26 (-0.08, 0.60)* | *0.26 (-0.02, 0.54)* | **0.40 ( 0.02, 0.77)** | **0.60 ( 0.25, 0.96)** | **0.31 (-0.07, 0.68)** |
| Age | *-0.03 (-0.14, 0.08)* | *0.07 (-0.04, 0.18)* | *0.02 (-0.07, 0.12)* | *0.02 (-0.06, 0.10)* | *0.06 (-0.05, 0.17)* | *0.07 (-0.03, 0.18)* | *0.05 (-0.06, 0.16)* |
| Smoking | *0.06 (-0.20, 0.32)* | -0.29 (-0.55, -0.03) | *-0.24 (-0.46, -0.02)* | *0.06 (-0.13, 0.26)* | *-0.23 (-0.49, 0.03)* | *-0.22 (-0.46, 0.02)* | *-0.25 (-0.51, 0.01)* |
| Alcohol | *0.17 (-0.11, 0.45)* | **0.32 ( 0.04, 0.60)** | *0.25 ( 0.00, 0.49)* | *0.11 (-0.10, 0.32)* | **0.35 ( 0.07, 0.63)** | **0.31 ( 0.04, 0.57)** | **0.33 ( 0.05, 0.61)** |
| **Wave 4 (Aged 23 – 28 yrs.)** | |  |  |  |  |  |  |
| SGA | **0.48 ( 0.16, 0.80)** | *-0.33 (-0.66, 0.00)* | *-0.27 (-0.56, 0.03)* | **-0.50 (-0.73, -0.26)** | *-0.30 (-0.63, 0.04)* | **-0.49 (-0.80, -0.17)** | *-0.14 (-0.46, 0.19)* |
| Female | **0.67 ( 0.42, 0.93)** | *0.22 (-0.05, 0.48)* | **1.17 ( 0.94, 1.41)** | **-1.41 (-1.61, -1.22)** | *0.20 (-0.07, 0.47)* | **-0.33 (-0.58, -0.08)** | **0.61 ( 0.34, 0.87)** |
| Urban | **-0.44 (-0.82, -0.07)** | **0.62 ( 0.23, 1.00)** | **0.36 ( 0.01, 0.71)** | **0.35 ( 0.07, 0.63)** | **0.49 ( 0.10, 0.87)** | **0.72 ( 0.35, 1.09)** | **0.36 (-0.02, 0.74)** |
| Age | *-0.06 (-0.17, 0.05)* | 0.14 ( 0.03, 0.25) | 0.10 ( 0.01, 0.20) | *0.01 (-0.07, 0.09)* | 0.13 ( 0.01, 0.24) | 0.14 ( 0.03, 0.25) | 0.12 ( 0.01, 0.23) |
| Smoking | *0.00 (-0.28, 0.28)* | *-0.21 (-0.50, 0.08)* | *-0.20 (-0.46, 0.05)* | *0.06 (-0.15, 0.27)* | *-0.22 (-0.51, 0.07)* | *-0.19 (-0.47, 0.09)* | *-0.23 (-0.52, 0.06)* |
| Alcohol | *-0.17 (-0.45, 0.11)* | *0.06 (-0.23, 0.35)* | *0.05 (-0.21, 0.30)* | *0.11 (-0.10, 0.32)* | *0.04 (-0.25, 0.34)* | *0.04 (-0.23, 0.32)* | *0.03 (-0.25, 0.32)* |
| **Wave 5 (Aged 29 – 36 yrs.)** | |  |  |  |  |  |  |
| SGA | *0.17 (-0.13, 0.48)* | *-0.28 (-0.59, 0.02)* | -0.29 (-0.57, -0.02) | **-0.49 (-0.70, -0.28)** | *-0.26 (-0.57, 0.05)* | **-0.46 (-0.75, -0.16)** | *-0.11 (-0.41, 0.20)* |
| Female | **0.50 ( 0.25, 0.74)** | *0.18 (-0.06, 0.42)* | **1.23 ( 1.02, 1.44)** | **-1.43 (-1.59, -1.26)** | *0.12 (-0.12, 0.37)* | **-0.37 (-0.60, -0.14)** | **0.50 ( 0.26, 0.74)** |
| Urban | *-0.19 (-0.54, 0.15)* | **0.42 ( 0.08, 0.75)** | *0.21 (-0.09, 0.51)* | **0.34 ( 0.10, 0.57)** | *0.35 ( 0.00, 0.69)* | **0.52 ( 0.20, 0.85)** | *0.25 (-0.09, 0.59)* |
| Age | *0.01 (-0.07, 0.09)* | *0.05 (-0.03, 0.13)* | *0.03 (-0.04, 0.11)* | *-0.02 (-0.07, 0.04)* | *0.05 (-0.03, 0.14)* | *0.05 (-0.03, 0.12)* | *0.05 (-0.03, 0.14)* |
| Smoking | *0.13 (-0.15, 0.40)* | **-0.54 (-0.81, -0.27)** | -0.29 (-0.53, -0.04) | *0.00 (-0.19, 0.19)* | *-0.45 (-0.73, -0.18)* | *-0.52 (-0.78, -0.27)* | **-0.43 (-0.70, -0.16)** |
| Alcohol | *-0.18 (-0.44, 0.07)* | *0.18 (-0.07, 0.43)* | *0.13 (-0.09, 0.35)* | *0.05 (-0.12, 0.22)* | *0.12 (-0.14, 0.37)* | *0.19 (-0.05, 0.43)* | *0.09 (-0.16, 0.34)* |
| Wave 2 models included sex, age, SGA status, and residential location.  Wave 3,4, and 5 models included sex, age, SGA status, residential location, smoking, and alcohol use.  To assist in observing stronger associations, figures in **bold** represent significant associations where the coefficient ≥ 0.30 (absolute). Figures *italicised* represent non-significant associations. | | | | | | | |

| **SUPPLEMENTAL TABLE S3:** Longitudinal analyses: Differences in anthropometric z-score marginal means between SGA and non-SGA (reference) estimated at 11, 18, 25, and 32 years of age, stratified by residential location among those who completed all study waves (n = 283). These are post-hoc contrasts from the linear mixed models with interaction terms between SGA and residential location. | | | | | | | |
| --- | --- | --- | --- | --- | --- | --- | --- |
| *Stratified* | **ABSI** | **BMI** | **Fat %** | **Height** | **Waist circ.** | **Weight** | **Waist/height ratio** |
| *By* | Difference (95%CI) | Difference (95%CI) | Difference (95%CI) | Difference (95%CI) | Difference (95%CI) | Difference (95%CI) | Difference (95%CI) |
| **Age 11** |  |  |  |  |  |  |  |
| Remote | *-0.02 (-0.30, 0.27)* | **-0.21 (-0.39, -0.03)** | **-0.29 (-0.56, -0.02)** | *-0.17 (-0.35, 0.01)* | **-0.21 (-0.40, -0.03)** | **-0.21 (-0.36, -0.05)** | *-0.20 (-0.41, 0.02)* |
| Urban | *0.57 (-0.18, 1.31)* | *-0.25 (-0.58, 0.08)* | *-0.27 (-0.89, 0.35)* | *-0.23 (-0.56, 0.10)* | **-0.27 (-0.69, 0.15)** | *-0.25 (-0.53, 0.03)* | *-0.08 (-0.58, 0.41)* |
| **Age 18** |  |  |  |  |  |  |  |
| Remote | **0.37 ( 0.07, 0.67)** | *-0.20 (-0.42, 0.02)* | *-0.26 (-0.53, 0.01)* | **-0.25 (-0.40, -0.10)** | *-0.13 (-0.35, 0.08)* | **-0.30 (-0.49, -0.11)** | *-0.02 (-0.27, 0.22)* |
| Urban | *0.20 (-0.48, 0.89)* | **-0.40 (-0.77, -0.03)** | **-0.62 (-1.19, -0.05)** | **-0.51 (-0.79, -0.24)** | **-0.44 (-0.84, -0.04)** | **-0.58 (-0.89, -0.26)** | *-0.26 (-0.73, 0.21)* |
| **Age 25** |  |  |  |  |  |  |  |
| Remote | **0.51 ( 0.18, 0.83)** | **-0.30 (-0.57, -0.02)** | *-0.23 (-0.50, 0.04)* | **-0.23 (-0.37, -0.09)** | *-0.17 (-0.43, 0.08)* | **-0.38 (-0.62, -0.15)** | *-0.06 (-0.36, 0.25)* |
| Urban | *-0.09 (-0.79, 0.61)* | *-0.16 (-0.62, 0.29)* | *-0.32 (-0.86, 0.21)* | **-0.51 (-0.77, -0.24)** | *-0.35 (-0.82, 0.11)* | *-0.38 (-0.77, 0.00)* | *-0.18 (-0.73, 0.37)* |
| **Age 32** |  |  |  |  |  |  |  |
| Remote | **0.41 ( 0.05, 0.78)** | *-0.29 (-0.63, 0.05)* | *-0.22 (-0.51, 0.06)* | **-0.25 (-0.40, -0.11)** | *-0.18 (-0.49, 0.13)* | **-0.40 (-0.69, -0.11)** | *-0.04 (-0.40, 0.33)* |
| Urban | *-0.38 (-1.25, 0.49)* | *0.04 (-0.55, 0.64)* | *0.01 (-0.64, 0.65)* | **-0.50 (-0.83, -0.17)** | *-0.30 (-0.91, 0.31)* | *-0.22 (-0.72, 0.29)* | *-0.11 (-0.84, 0.61)* |
| Models included sex, age, SGA status, and residential location.  To assist in observing stronger associations, figures in **bold** represent significant differences ≥ 0.20 (absolute). Figures *italicised* represent non-significant differences. | | | | | | | |

| **SUPPLEMENTAL TABLE S4:** Attrition analyses: Mean anthropometric measures at the current wave, comparing those who participated in the following wave versus those who did not. P values are from two-sided T-Tests. | | | | | | | | | |
| --- | --- | --- | --- | --- | --- | --- | --- | --- | --- |
|  | **Wave 2 to Wave 3** | | | **Wave 3 to Wave 4** | | | **Wave 4 to Wave 5** | | |
|  | Wave 2 Only | Wave 2 & 3 | *P value* | Wave 3 Only | Wave 3 & 4 | *P value* | Wave 4 Only | Wave 4 & 5 | *P value* |
| ABSI | 0.082 | 0.082 | 0.643 | 0.079 | 0.080 | 0.418 | 0.082 | 0.082 | 0.234 |
| BMI | 17.0 | 16.9 | 0.781 | **22.9** | **21.2** | **0.017** | 24.7 | 23.4 | 0.108 |
| FAT% | 21.3 | 21.3 | 0.971 | 22.5 | 20.1 | 0.107 | 25.4 | 24.6 | 0.599 |
| Height (cm) | 145.3 | 143.3 | 0.054 | 168.7 | 167.6 | 0.282 | 169.2 | 167.5 | 0.109 |
| Waist circ. (cm) | 65.2 | 64.4 | 0.402 | **82.4** | **78.5** | **0.048** | **90.1** | **85.4** | **0.016** |
| Weight (kg) | 36.7 | 35.6 | 0.327 | **65.9** | **59.8** | **0.015** | **71.2** | **65.9** | **0.040** |
| Weight/Height ratio | 0.4 | 0.4 | 0.940 | 0.5 | 0.5 | 0.083 | 0.5 | 0.5 | 0.058 |

| 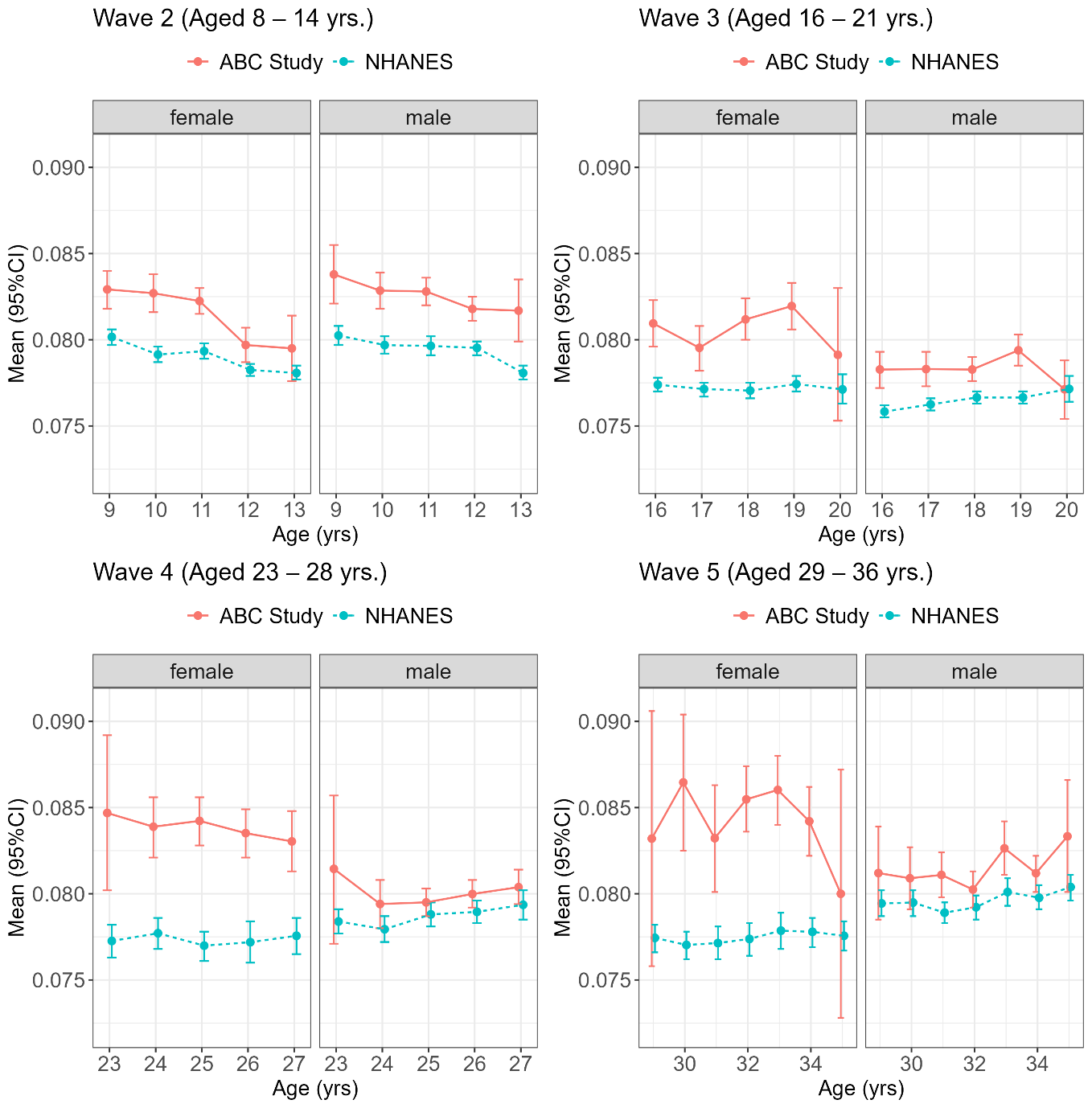 |
| --- |
| **SUPPLEMENTAL FIGURE S1:** Mean (95% CI) ABSI scores within ages comparing the original NHANES data used to develop ABSI (1) and ABC study data within each study wave (linked to NHANES by age), stratified by gender. Data were restricted to where the age in each ABC wave has ≥ 5 participants. |
